# Diagnosis of secondary tuberculosis infection in an asymptomatic elderly with cancer using next-generation sequencing: Case report

**DOI:** 10.1515/biol-2025-1123

**Published:** 2025-06-12

**Authors:** Jiahuan Huang, Wenbo Ren, Weishang Hu, Jianfang Ni

**Affiliations:** Geriatric Medicine Center, Department of Geriatric Medicine, Zhejiang Provincial People’s Hospital, Affiliated People’s Hospital, Hangzhou Medical College, Hangzhou, Zhejiang, China; Center for Rehabilitation Medicine, Rehabilitation & Sports Medicine Research Institute of Zhejiang Province, Department of Rehabilitation Medicine, Zhejiang Provincial People’s Hospital, Affiliated People’s Hospital, Hangzhou Medical College, Hangzhou, Zhejiang, China; Geriatric Medicine Center, Department No. 2 of Acupuncture & Massage, Zhejiang Provincial People’s Hospital, Affiliated People’s Hospital, Hangzhou Medical College, Hangzhou, Zhejiang, China

**Keywords:** metagenomic next-generation sequencing, *Mycobacterium tuberculosis*, cancer, elderly

## Abstract

In recent years, there has been a notable increase in the prevalence of tumors and tuberculosis (TB), particularly among elderly and immunocompromised populations. Early diagnosis and treatment are crucial for significantly improving patient outcomes. However, traditional diagnostic methods exhibit certain limitations. The rapid advancement of metagenomic next-generation sequencing (mNGS) has shown promising applications in the field of infectious diseases. We describe an 88-year-old male with multiple comorbidities, including newly diagnosed localized prostate cancer, who presented asymptomatically. Routine mNGS screening unexpectedly identified *Mycobacterium tuberculosis*, suggesting that malignancy may foster immune conditions favoring latent TB reactivation. This case emphasizes mNGS’s role as a rapid, sensitive diagnostic adjunct for occult infections in high-risk populations.

## 1 Introduction

Tuberculosis (TB) and tumors represent major global health burdens associated with high morbidity and mortality rates, posing substantial public health concerns that severely threaten human health. As the leading infectious cause of death worldwide [1]. TB demonstrates a complex bidirectional relationship with malignancies. Numerous epidemiological studies have demonstrated the coexistence of TB and tumors, with TB acting as a predisposing factor for tumor development, while patients with tumors face an increased risk of TB [1,2]. Clinical studies further establish active malignancies as independent risk factors for *Mycobacterium tuberculosis* (MTB) infections [3], underscoring the critical importance of early diagnosis and treatment for improving patient prognosis. Current diagnostic approaches for latent TB infection, including interferon-γ release assays and tuberculin skin testing, are compromised by notable limitations that may lead to misdiagnosis and subsequent clinical management challenges [4]. It is therefore crucial to develop rapid screening methodologies to guide clinical decision-making during initial disease stages. The recent advent of metagenomic next-generation sequencing (mNGS) has demonstrated significant diagnostic potential across infectious diseases [5,6]. We present a clinically challenging case of asymptomatic MTB detection via mNGS in an elderly patient with multiple comorbidities, including newly diagnosed localized prostate cancer.

## 2 Case presentation

**Figure 1:**
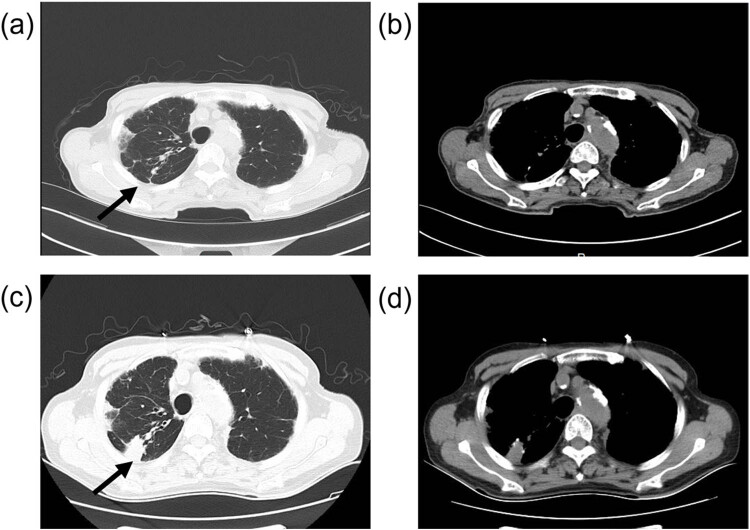
The patient's chest CT scan results from various time points revealed the emergence of new patchy high-density shadows, as indicated by the black arrow, in the upper lobe of the right lung (c) and (d), compared to previous scans (a) and (b).


An 88-year-old male was admitted to our hospital in April 2024 with symptoms of fever, cough, and sputum production. Following laboratory tests, pneumonia was suspected, leading to the administration of antibiotics, expectorants, cough suppressants, and intravenous fluids. The patient’s symptoms significantly improved, and he was subsequently hospitalized for an extended period. Routine evaluations demonstrated clinical stability until August 2024 when he developed mild asthenia without concomitant symptoms. The patient’s medical history included newly diagnosed prostate cancer (diagnosed 2 months prior) managed with androgen deprivation therapy (goserelin co-administered with bicalutamide). Serial monitoring revealed stable prostate-specific antigen levels, with comorbidities encompassing chronic kidney disease and congestive heart failure. Physical examination identified bilateral coarse breath sounds with scattered rhonchi and rales on pulmonary auscultation. Cardiovascular and abdominal assessments were unremarkable. Standard laboratory parameters remained within normal ranges. Sputum was collected for mNGS, which subsequently confirmed the presence of a Mycobacterium TB infection. A subsequent chest computed tomography imaging demonstrated bilateral lower lobe parenchymal opacities suggestive of inflammatory etiology, alongside bilateral fibroproliferative changes predominating in the right upper lobe, radiologically consistent with tuberculous involvement (Figure 1). The patient was ultimately diagnosed with TB and subsequently received appropriate treatment. At a follow-up 3 months later, after receiving standardized treatment, the patient exhibited a negative sputum culture result and demonstrated significant improvement.

**Informed consent:** Informed consent has been obtained from all individuals included in this study.
**Ethical approval:** The research related to human use has been complied with all the relevant national regulations and institutional policies and in accordance with the tenets of the Helsinki Declaration and has been approved by the Ethics Committee of the Zhejiang Provincial People’s Hospital.

## 3 Discussion

TB and cancer constitute dual global health burdens with significant morbidity and mortality. Epidemiological studies confirm a bidirectional pathophysiological interplay between these diseases: TB not only potentiates tumorigenesis but also facilitates TB reactivation in cancer patients [7]. This symbiosis stems from cancer-induced multifactorial immunosuppression, which independently predisposes to active MTB infection [2]. Potential mechanisms through which tumors may induce TB include tumor effects, as well as chemotherapy and immunotherapy [8]. However, the associated risk varies considerably among different cancer types, with lung cancer and hematologic malignancies exhibiting higher susceptibility [3].

The patients described in this study have not undergone chemotherapy or immunotherapy. Consequently, TB may be induced by the effects of tumors; specifically, the metabolic changes in tumor cells driven by oncogenes may compromise the body’s immune response by altering the tumor microenvironment, thereby causing significant adverse effects on normal immune defense functions [9]. However, the precise mechanism by which tumors induce TB remains inadequately understood.

Challenges in diagnosing TB persist, particularly among immunocompromised populations. Although traditional pathogenic microorganism detection techniques are widely employed in clinical settings, they exhibit limitations such as low positive rates and extended detection periods, complicating the accurate identification of strains and increasing the risk of clinical misdiagnosis, which can severely affect patient prognosis [10]. However, with the rapid advancement of laboratory diagnostic techniques, the detection rate of MTB has significantly improved, rendering traditional diagnostic methods insufficient to meet clinical needs. mNGS is an innovative technology increasingly utilized for detecting infectious sources, as evidenced by its unique advantages outlined below. One of the primary advantages of mNGS is its high sensitivity, which results from its capacity to sequence DNA at sufficient depth, thereby enabling the accurate detection of pathogens even when present in low quantities. Additionally, mNGS is beneficial for diagnosing mixed infections involving NTM alongside other pathogens. Furthermore, compared to the average feedback time of 7–14 days required for traditional culture-based methods, the rapid turnaround time of 2–3 days for obtaining pathogenic evidence through mNGS significantly enhances early and prompt disease diagnosis in clinical practice [11].

Despite its transformative potential, mNGS has notable limitations. While mNGS exhibits high sensitivity and can theoretically identify all pathogens with known genomic sequences – over 8,000 cataloged species – this broad-spectrum detection also heightens the risk of false positives, which can arise from environmental or technical contamination as well as interference from normal microbiota. Such interference may obscure true pathogens amidst background signals. Additionally, the high cost of mNGS constitutes a significant limitation, primarily due to the expensive equipment and operational expenses associated with its use. The analysis of blood, cerebrospinal fluid, and respiratory specimens via mNGS incurs costs ranging from $1,000 to $2,500 per test [12]. Furthermore, this technology relies on advanced technical infrastructure, including sequencing platforms and bioinformatics pipelines [10].

It is noteworthy that mNGS demonstrates varying diagnostic utility for different mycobacterial infections. According to the literature, mNGS plays distinct roles in differentiating non-tuberculous *mycobacterial* pulmonary diseases (NTM-PD) from drug-resistant tuberculosis (DR-TB). For NTM-PD, mNGS excels at rapidly identifying diverse NTM species (e.g., *Mycobacterium avium* complex, *M. abscessus*) directly from clinical samples, thereby bypassing the need for culture-dependent methods and facilitating species-specific treatment decisions by detecting environmental *mycobacteria* with intrinsic drug resistance profiles [13]. In contrast, for DR-TB, mNGS focuses on detecting the MTB complex and its drug-resistance mutations (e.g., rpoB for rifampicin resistance) to guide tailored antitubercular regimens [14].

Overall, our findings demonstrate that mNGS enhances precision medicine by rapidly and accurately identifying pathogens. This capability facilitates the earlier optimization of targeted therapies, which is particularly critical for immunocompromised patients who are vulnerable to polymicrobial and atypical infections, especially in cases where conventional diagnostics fail. Currently, most guidelines cite recommendations for routine screening for latent TB based on individual patient assessment [13].

## 4 Conclusion

As a revolutionary detection method, mNGS offers significant advantages in identifying pathogens associated with infectious diseases. This capability supports its adoption as a critical first-line diagnostic tool for immunocompromised patients, thereby facilitating the optimization of therapeutic interventions.
